# A labelled image dataset of genuine and counterfeit Ugandan banknotes

**DOI:** 10.1016/j.dib.2026.113293

**Published:** 2026-09-26

**Authors:** Denish Azamuke, Calvin Bamwesigye Kiiza, Marriette Katarahweire, Engineer Bainomugisha

**Affiliations:** Department of Computer Science, Makerere University, Plot 56 Pool Road, P. O. Box 7062, Kampala, Uganda

**Keywords:** Banknote authentication, Counterfeit detection, Currency image analysis, Computer vision, Machine learning

## Abstract

This data article describes a labelled image dataset of genuine and counterfeit Ugandan banknotes prepared for research on visual currency authentication and machine-learning-based counterfeit detection. The dataset was compiled from smartphone photographs of banknotes in the denominations of UGX 1000, 2000, 5000, 10,000, 20,000, and 50,000, and curated into a single corpus labelled as genuine or counterfeit. The banknotes, especially counterfeit ones, were obtained from mobile money agent shops, micro-finance institutions, banks, and local markets in Uganda. Exact duplicate image files were identified using SHA-256 content hashes. Redundant copies and files that could not be decoded after copying or conversion were excluded from the clean corpus. The clean dataset contains 1426 image files, comprising 666 genuine and 760 counterfeit banknote images. This article describes the master image corpus, organized into genuine and counterfeit banknotes, and its denomination distribution. This pilot-scale dataset can be used for exploratory research, prototyping, and teaching image classification methods for banknote authenticity screening.'

Specifications Table

**Table:** 

Subject	Data Science
Specific subject area	Applied machine learning and computer vision for banknote authentication
Type of data	Raw and curated image files; JPEG and PNG images.
Data collection	Images of UGX 1000, 2000, 5000, 10,000, 20,000, and 50,000 banknotes were captured using smartphone cameras with default camera settings under everyday ambient lighting conditions. Genuine and counterfeit notes were gathered from mobile money agent shops, micro-finance institutions, banks, and local markets in Uganda, then curated into a cleaned, labelled corpus. The original data collection was conducted in March 2024 by four photographers.
Data source location	Institution: Department of Computer Science, Makerere University. City/Town/Region: Kampala, Uganda. Physical banknotes were sourced from mobile money agent shops, micro-finance institutions, banks, and local markets in Uganda.
Data accessibility	Repository name: Mendeley Data Data identification number: doi:10.17632/dz9j2hbd47.1 Direct URL to data: https://data.mendeley.com/datasets/dz9j2hbd47/1
Related research article	None.

## 1 Value of the Data

• The dataset provides images of genuine and counterfeit Ugandan banknotes across six denominations, supporting research on the visual characteristics of Uganda’s currency. Counterfeit notes were sourced from financial institutions and other cash-handling environments, providing examples encountered in circulation rather than digitally generated counterfeits.
• As a pilot-scale resource of 1426 images, the dataset supports exploratory classification, transfer-learning experiments, and teaching. It offers a starting point for developing Ugandan banknote screening methods.
• Images captured with smartphone cameras under everyday ambient light provide material for investigating sensitivity to image quality, background, and capture conditions. Researchers can use these variations to examine whether models rely on contextual cues rather than features of banknote authenticity.
• The per-image denomination class counts support analysis of denomination imbalance and the design of further data collection. The documented curation process and SHA-256 hashes support reproducible file checks, while the disclosed repeated views provide a starting point for investigating specimen identity before evaluating generalisation to unseen banknotes.

## 2 Background

Counterfeit currency detection is a practical visual-recognition problem since authentic and counterfeit notes can differ in texture, print quality, color response, serial markings, security features, and visual appearance under different lighting conditions. Image datasets enable evaluation of discriminative pattern learning. The dataset [1] was compiled to support this type of work for Ugandan banknotes across the six common denominations of UGX 1000, 2000, 5000, 10,000, 20,000, and 50,000.

Counterfeit banknotes were collected directly from financial institutions and other cash-handling environments in Uganda, rather than being generated from images of genuine notes. This approach enriches the dataset with unique counterfeit samples and distinguishes our work from related studies that generate counterfeit samples from previously collected images of genuine banknotes [2]. Other studies [[2], [3], [4]] on counterfeit banknote detection have relied on limited datasets.

The dataset was subjected to a systematic curation and integrity-verification process to enhance its reliability. Exact duplicate images were identified using SHA-256 [5] content hashes, and redundant file instances were removed from the curated corpus. Image files that could not be decoded following format conversion were excluded. The resulting dataset retained the original class labels while providing a cleaned corpus for exploratory model development and evaluation.

## 3 Data Description

This article mainly describes the master corpus in the all_images directory of labelled_ugandan_banknote_dataset_clean [1]. It contains 1426 labelled images in genuine and counterfeit subfolders, as summarized in Table 1.

**Table 1:** Class composition of the cleaned, labelled banknote image corpus.

Class	Image count	Share of clean corpus
Counterfeit	760	53.3%
Genuine	666	46.7%
Total	1426	100.0%

The 1426 retained files comprise 1424 JPEG images and 2 PNG images. Image resolutions range from 480 × 270 pixels (0.13 megapixels) to 3456 × 4608 or 4608 × 3456 pixels (15.93 megapixels), with a median of 12.19 megapixels. These are measured file dimensions, not nominal camera sensor resolutions.

Camera-model metadata for the images includes SM-G781V, HUAWEI NXT-L29, A063, and SM-A217F. These identifiers are reported exactly as stored during data collection.

Both front (obverse) and back (reverse) views occur in the dataset, but both sides were not photographed for every banknote. The corpus is therefore not a collection of consistently paired front/back images. Filenames identify individual image files; they do not establish which front and back views belong to the same physical banknote.

The image collection covered Ugandan banknote denominations of 1000, 2000, 5000, 10,000, 20,000, and 50,000 shillings. The corpus includes images of clean, old, dirty, and visibly used notes, reflecting the range of conditions encountered during everyday cash handling. Table 2 reports image counts by denomination and authenticity class.

**Table 2:** Image counts by primary banknote denomination and authenticity class.

Denomination (UGX)	Counterfeit	Genuine	Total
1000	18	154	172
2000	71	78	149
5000	246	55	301
10,000	264	257	521
20,000	102	110	212
50,000	59	12	71
**Total**	**760**	**666**	**1426**

Counts refer to image files, not independently identified physical banknotes. Each image was counted once using the denomination of its main foreground banknote; additional background notes in the same image were not counted separately. Authenticity classes were inherited from classification by senior bankers using a common light-based currency verification machine in commercial banks.

However, the splits directory stores the same included images in train_set, validation_set, and test_set subfolders, each with genuine and counterfeit subdirectories. The composition of images in these subfolders is shown in Table 3, with 998 images in the training set and 214 in the validation and test sets.

**Table 3:** Stratified train, validation, and test splits.

Split	Counterfeit	Genuine	Total
Train	532	466	998
Validation	114	100	214
Test	114	100	214

Fig. 1 shows examples of clean genuine and counterfeit banknote images from the curated corpus, including different denominations of UGX 2000, 5000, 10,000, and 20,000.

**Fig. 1:**
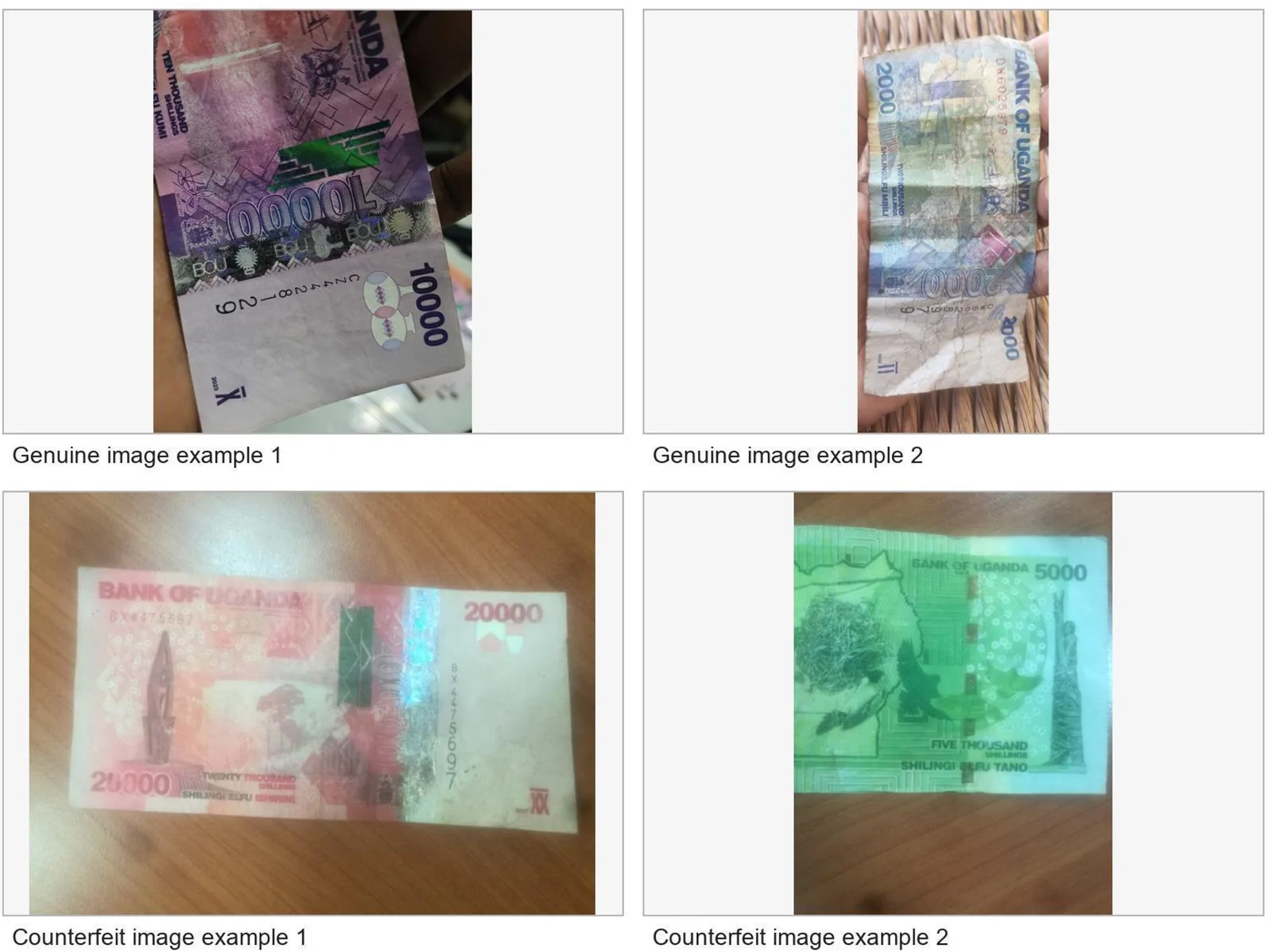
Representative examples of genuine and counterfeit images of Ugandan banknotes.

## 4 Experimental Design, Materials and Methods

The original image collection was conducted in March 2024 by four photographers. Images of Ugandan banknotes were initially gathered from mobile money agent shops, micro-finance institutions, banks, and local markets in Uganda. Physical banknotes in the denominations of UGX 1000, 2000, 5000, 10,000, 20,000, and 50,000 were photographed using smartphone cameras with default camera settings under everyday ambient lighting conditions. The image files were then transferred to a laptop for cleaning and verification so that duplicate files, unsupported formats, and files of uncertain origin could be identified, corrected where possible, or removed from the clean corpus. Subsequent visual review identified repeated views of some banknotes, including differences in framing, focus, and camera position. No separate identifiers were assigned to the physical banknotes; the standardized filenames identify image files rather than independently recorded physical specimens. Both genuine and counterfeit notes were photographed under ambient light only; thus, the banknote inspection device did not provide illumination during image capture.

Three senior bankers assessed each banknote using the common light-based banknote inspection machines to classify it as genuine or counterfeit. The bankers permitted photography only of notes whose classification they were certain of after checking; notes whose authenticity remained uncertain were not photographed for the dataset. The machine models and light sources were not confirmed; however, it was the common light-based banknote verification machine used in African markets. Counterfeit notes included examples retained by financial institutions. These two categories were maintained during image capture and storage by placing the files in separate genuine and counterfeit folders. Therefore, the original class labels by three senior bankers were preserved throughout the data capture and curation process. Old, dirty, and visibly used notes were included to represent the physical variation encountered during routine cash circulation.

Image resolution and visible detail vary across the retained files; fine print and security features are not equally discernible in all photographs. No counterfeit samples were produced from genuine-note images during data collection.

Files were scanned recursively from the source folders only when they were located under a genuine or counterfeit subfolder and had one of the following image extensions: .jpg, .jpeg, .png, and .heic. The curation workflow did not modify the original source folders.

For the denomination inventory, all 1426 retained images were visually reviewed with assistance from OpenAI Codex. Local optical character recognition was used as a secondary check. Difficult readings and disagreements were inspected at higher magnification. A per-image index records the assigned denomination, original authenticity class, filename, and SHA-256 hash. The denomination counts were reconciled with the authenticity-class totals and the complete master corpus. All retained images were also screened for repeated views using perceptual image similarity and repeated serial-number readings. Candidate matches were assessed visually using distinctive wear, folds, markings, and scene details; matching serial numbers alone were not treated as proof of specimen identity.

For each image file, a SHA-256 [5] content hash was computed. This identified byte-identical files independently of filename, folder location, or file-copy history. Hashing was used to detect byte-identical duplicate files and duplicate groups with conflicting class labels. However, this does not detect visually similar images or establish the identity of a physical banknote.

The hashing step can be reproduced with the following Python code in Listing 1.

**Table:** 

import hashlib
def sha256_file(path):
digest = hashlib.sha256()
with open(path, "rb") as file:
for chunk in iter(lambda: file.read(1024 * 1024), b""):
digest.update(chunk)
return digest.hexdigest()

Listing 1. Python implementation for computing SHA-256 content hashes of image files.

Files with identical SHA-256 hashes were grouped as exact duplicates. Duplicate groups with conflicting labels would be excluded, although no label-conflict duplicate groups were detected. For duplicate groups with a single label, one canonical file was retained. The canonical record was chosen deterministically to make the preprocessing workflow reproducible.

HEIC files were tested for JPEG conversion. During transfer to the laptop, 72 files were reported to have been damaged and were unreadable during subsequent processing; these files were excluded after copying or conversion attempts. A subsequent integrity check of all 1426 retained images, comprising 760 counterfeit and 666 genuine images, completed both file verification and full pixel decoding using Pillow without errors. This confirms the readability of the retained corpus, but does not establish the cause of damage to the excluded files. The curation and verification outcomes are summarized in Table 4.

**Table 4:** Curation and verification checks used to prepare the clean dataset.

Curation check	Result
Input image files scanned	1519
Clean included images	1426
Exact duplicate file instances excluded	21
Unreadable file instances excluded after copy/conversion	72

## Limitations

With 1426 images across two authenticity classes and six denominations, this is a pilot-scale dataset. Its limited size and uneven denomination coverage constrain the diversity of examples available for model development and evaluation. It supports exploratory research and transfer-learning experiments, but is insufficient on its own to establish a general-purpose banknote authentication benchmark or demonstrate reliable performance in deployment. Thus, a larger and more diverse collection is required for an independent evaluation. Excluding 72 files that became unreadable after transfer may have affected dataset coverage; successful decoding of the retained images does not rule out selection effects from these exclusions.

Both genuine and counterfeit notes were photographed under ambient light only using smartphone cameras. Lighting, shadows, background, focus, orientation, glare, and camera angle vary across images. Differences between cameras and their image processing may also affect colour and detail. Ambient-light photography does not by itself ensure equivalent illumination across authenticity classes. Lighting, background, and device characteristics may therefore provide cues unrelated to banknote authenticity, and their contribution should be assessed before interpreting model performance.

The authenticity labels reflect assessments by three senior bankers using light-based banknote inspection machines, with photography limited to notes they classified with certainty. However, the machine models and light sources have not been confirmed, and the available documentation does not establish that assessments were independent or quantify agreement between assessors or label error. The labels should therefore be interpreted in the context of this verification procedure.

The corpus includes repeated views of some physical banknotes, with differences in framing, focus, and camera position. SHA-256 deduplication removes byte-identical files but does not remove every repeated view or establish physical-specimen identity. No specimen identifiers were recorded during collection, and the total number of distinct physical banknotes has not been established. The 1426 images should therefore not be interpreted as 1426 independent banknotes. Users evaluating generalization to unseen banknotes should establish specimen groups before designing their evaluation.

## Ethics Statement

Access to banknotes was arranged with participating banks and other cash-handling parties under a non-disclosure agreement that permitted access, academic research, and publication of the banknote photographs. The banknotes were mainly photographed at the banks and remained in bank custody throughout; researchers did not remove or retain the physical notes. The published dataset is intended solely for academic research on banknote authentication.

Besides, the current dataset does not involve human subjects, animal experiments, or data collected from social media platforms. The images describe banknotes and do not contain intentionally collected personal information.

## Declaration of Generative AI and AI-Assisted Technologies in the Writing Process

During the preparation of this article, the authors used OpenAI Codex to improve grammar and readability and to assist with visual denomination annotation and count reconciliation. After using this tool/service, the authors reviewed and edited the content as needed and take full responsibility for the content of the publication.

## CRediT authorship contribution statement

**Denish Azamuke:** Conceptualization, Methodology, Validation, Writing – original draft, Writing – review & editing. **Calvin Bamwesigye Kiiza:** Data curation, Methodology, Writing – review & editing. **Marriette Katarahweire:** Project administration, Writing – review & editing. **Engineer Bainomugisha:** Writing – review & editing, Supervision.
